# Novel study design to assess the efficacy and tolerability of antiseizure medications for focal‐onset seizures in infants and young children: A consensus document from the regulatory task force and the pediatric commission of the International League against Epilepsy (ILAE), in collaboration with the Pediatric Epilepsy Research Consortium (PERC)

**DOI:** 10.1002/epi4.12356

**Published:** 2019-09-04

**Authors:** Stéphane Auvin, Jacqueline French, Denis Dlugos, Kelly G. Knupp, Emilio Perucca, Alexis Arzimanoglou, Ed Whalen, Renée A. Shellhaas

**Affiliations:** ^1^ Department of Pediatric Neurology AP‐HP Robert‐Debré University Hospital CRMR Epilepsies Rares Paris France; ^2^ Université de Paris INSERM U1141 Paris France; ^3^ NYU Langone School of Medicine New York NY USA; ^4^ Division of Neurology Children's Hospital of Philadelphia (CHOP) Philadelphia PA USA; ^5^ Departments of Neurology and Pediatrics Perelman School of Medicine at the University of Pennsylvania Philadelphia PA USA; ^6^ Department of Pediatrics and Neurology University of Colorado Aurora CO USA; ^7^ Department of Internal Medicine and Therapeutics Member of the ERN EpiCARE University of Pavia and IRCCS Mondino Foundation Pavia Italy; ^8^ Department of Clinical Pediatric Epileptology and Functional Neurology Member of the ERN EpiCARE University Hospitals of Lyon (HCL) Lyon France; ^9^ Global Biometrics and Data Management Pfizer Inc New York NY USA; ^10^ Division of Pediatric Neurology Department of Pediatrics Michigan Medicine Ann Arbor MI USA

**Keywords:** antiseizure drugs, children, clinical trials, drug development, infants

## Abstract

High‐quality placebo‐controlled drug trials for focal‐onset seizures in infants and children younger than 4 years have become increasingly difficult to perform because of eligibility constraints and onerous study designs. Traditional designs used in these populations require a high baseline seizure frequency, two hospitalizations for video‐electroencephalography (video‐EEG) monitoring, and willingness to accept potential exposure to placebo when the drugs to be tested are usually already available for off‐label prescription. To address these constraints, the International League Against Epilepsy (ILAE) regulatory taskforce and the ILAE pediatric commission, in collaboration with the Pediatric Epilepsy Research Consortium (PERC), propose a novel trial design which involves seizure counting by caregivers based on previous video‐EEG/video validation of specific seizure semiologies. We present a novel randomized placebo‐controlled trial design intended to be used for studying new antiseizure medications (ASMs) for focal‐onset seizures (FOS) in children aged one month to four years. This design uses “time to Nth seizure” as the primary outcome and incorporates a new element of variable baseline duration. This approach permits enrollment of infants with lower seizure burden, who might not have video‐EEG‐recorded seizures within 2‐3 days of monitoring. Repeated hospitalizations for video‐EEG recordings are avoided, and duration of baseline and exposure to placebo or ineffective treatment(s) are minimized. By broadening eligibility criteria, reducing risks from prolonged placebo exposure, and relying on validated recording of seizure counting by caregivers, clinical trials will be likely to be completed more efficiently than in the recent past.

**Key Points**

- The traditional infantile placebo‐controlled design for focal‐onset seizures has become impracticable.
- We propose a novel trial design which involves seizure counting by caregivers based on previous video‐EEG/video validation of specific seizure semiologies.
- The baseline duration is adjusted based on individual seizure burden, and duration of treatment is linked to seizure response using a time‐to‐event endpoint.

## 1 INTRODUCTION

Focal‐onset seizures (FOS) are possibly the most ubiquitous seizure type, are a feature of the majority of epilepsy syndromes, and can occur from birth to old age.^1^ In the last several decades, there have been numerous new antiseizure medications (ASMs) approved for use in adults with FOS. Once drugs are approved in adults for this indication, it is important to determine whether they could also be valuable for children, particularly for those whose seizures remain resistant to available medications.^2^, ^3^

The US Pediatric Research Equity act (PREA), which was signed into US law in 2003, requires sponsors who receive an approval for a specific condition to perform studies that include children of all ages with the same condition, unless the requirement is waived or deferred.^4^ Similarly, in 2006, the European Union established that, for all drugs under development, a pediatric investigation plan (PIP) should be submitted to the European Medicine Agency (EMA) “not later than upon completion of the human pharmacokinetic studies in adults,” unless conditions apply for waiver or deferral.^5^

Since 2010, the EMA guideline on clinical investigation of medicinal products in the treatment of epileptic disorders accepted that efficacy of an ASM for FOS, if demonstrated in adults, can be extrapolated down to the age of four years “provided the dose is established” through appropriate pharmacokinetic and safety studies in children.^6^ Similarly, in 2017, in response to the “PEACE” (Pediatric Epilepsy Academic Consortium for Extrapolation) initiative,^7^ the FDA concluded that efficacy for FOS can be extrapolated from adult trials down to the age of four years, without the need for an additional efficacy trial.^8^ These regulatory developments were welcomed by the epilepsy community, because they allowed new ASMs to reach and benefit children more quickly. Yet, whether FOS in infants and very young children (under the age of 4 years) are similar enough to those of older children to allow extrapolation of effect remains a point of discussion. Importantly, ASM trials that focus on young children (<4 years old) and infants (>1 month old) are mandated by law (trials are waived in neonates up to one month old) and are urgently needed to provide safe clinical care to this population.^4^

The traditional placebo‐controlled design that has been used in this age‐group has become impracticable, for several practical and scientific reasons. Many clinicians and investigators find it difficult to justify enrolling an infant into these trials, since they require a very high baseline seizure frequency in order to limit duration of the baseline and treatment assessment phases, two hospitalizations for video‐electroencephalography (video‐EEG), and the risk of randomization to placebo, particularly when the drugs to be tested by this design are available off label by prescription in most countries. Families are reluctant to participate for these same reasons. Child neurologists are also reluctant to wait, conscious of the evidence‐based fact that persistence of epileptic seizures particularly at this age may further jeopardize neurodevelopment. Numerous ongoing studies are stalled, leading to a void of rigorous data available to use for the treatment of children with early‐life epilepsies. Moreover, by the time these studies are completed, the data may be substantially less useful, since the drugs will have been available commercially for a decade or more.

We present a novel randomized placebo‐controlled trial design intended to be used for studying new ASMs for FOS in children aged one month to four years. This design uses “time to Nth seizure” as the primary outcome and incorporates a new element of variable baseline duration. Both of these elements are intended to shorten the time until the child receives effective therapy. There is an urgent need for this design, as trials in this age‐group have been stalled for the last decade.^9^, ^10^

## 2 PREVIOUS INFANT ASM TRIALS

Since 1988, a total of 6 controlled trials of ASMs for the adjunctive treatment of FOS in infants have been completed.^11^, ^12^, ^13^, ^14^, ^15^, ^16^ Topiramate (TPM) and lamotrigine (LTG) were evaluated in infants aged one to 24 months.^11^, ^13^ while gabapentin (GBP), levetiracetam (LEV), oxcarbazepine (OXC), and pregabalin (PGB) were evaluated in children aged >1 month to <4 years.^12^, ^14^, ^15^, ^16^ The studies evaluating GBP, TPM, LTG, LEV, and PGB were placebo‐controlled, and the OXC study compared low/high dose groups. Ability to enroll has changed dramatically over time; the 1988 GBP study completed enrollment in four months, while the 2014‐2018 PGB study took ten times as long (42 months) to complete enrollment, supporting that this study design has become less practicable.^16^

Except for the LTG study (see below),^13^ studies conducted to date used FOS on EEG as the primary outcome. EEG protocols varied. FOS on continuous 48‐ to 72‐hour outpatient EEG were the primary outcome in the GBP study, and FOS on continuous 48‐ to 72‐hour inpatient video‐EEG were the primary outcome in the TPM, LEV, OXC, and PGB trials. Studies using EEG for inclusion required at least two FOS seizures on the baseline EEG. In fact, randomized patients had a higher FOS burden than required, with baseline median EEG seizure frequency ranging from 6 to 15 per day.^11^, ^12^, ^13^, ^14^, ^15^, ^16^

Electroencephalography (EEG) interpretation can lead to significant variability and requires clear criteria when used as an outcome measure. When clearly defined in the published studies (TPM, LEV),^11^, ^14^ FOS were required to have a minimum duration of ten seconds, involve at least two adjacent EEG electrodes, display clearly focal or asymmetric changes, and have a recognizable evolution on EEG. The TPM trial required that all EEG seizures used for study purposes have a corresponding clinical semiology.^11^ The LEV trial required clinical manifestations for all EEG seizures in patients six months to <4 years, but subclinical seizures were permitted in patients 1‐<6 months of age.

The LTG study was a responder‐enriched design. Patients who had responded to LTG during an initial open‐label phase were randomized to double treatment for up to 8 weeks with continued LTG or a switch from LTG to placebo. The LTG trial utilized seizure diaries for the primary efficacy endpoint, though baseline seizures were defined by EEG.^13^

In the GBP, TPM, and LTG studies,^11^, ^13^, ^15^ active treatment did not result in a significant reduction in FOS during the double‐blind comparison phase. The LEV, OXC, and PGB studies did show a significant reduction in FOS during the double‐blind comparison phase.^12^, ^14^, ^16^

## 3 PROPOSAL FOR A TIME‐TO‐EVENT CLINICAL TRIAL PROTOCOL

To address the issues outlined above, we present a novel trial protocol that is designed to adjust baseline duration and exposure to placebo or ineffective treatment based on the subject's seizure burden. Sample inclusion and exclusion criteria are presented in Table 1.

**Table 1:** Sample inclusion and exclusion criteria for an infant time‐to‐event randomized clinical trial of antiseizure medications for focal‐onset seizures

Inclusion criteria	Exclusion criteria
Male or female from ≥44 wk postmenstrual age to <4 y of age	Exclusively febrile seizures^b^
Diagnosis of epilepsy with FOS	Current seizures defined on EEG by electrodecrements, generalized paroxysmal fast activity, or generalized spike and wave^c^
Clinical evidence of *observable* FOS	Current hypsarrhythmia
Consistent semiology recorded on video‐EEG in the past 6 mo, or convincing video evidence of FOS with supportive interictal EEG and clinical data	Nonepileptic paroxysmal events that could be confused with seizures
Stable treatment regimen of 1‐2 medications at steady state^a^	Medical conditions or concomitant medications that cause undue safety risk or that may adversely influence compliance or data interpretation
	Progressive central nervous system disease

Abbreviation: FOS, Focal‐onset seizures.

a Stable treatment regimen defined as no new medications added, no change in antiseizure medication (ASM) dosage for ≥4 half‐lives, no ASM dose reduction by >20% within 2 wk of enrollment; ketogenic diet ratio stable for ≥3 mo; vagal nerve stimulator parameters stable for ≥6 mo.

b A history of febrile seizures in a child with current unprovoked FOS is acceptable.

c For example, epileptic spasms, tonic, atonic, or myoclonic seizures.

Seizures with clinical signs will be confirmed by video‐EEG (preferred) or video (with clear semiology and supporting clinical and interictal EEG data) for each study participant and reviewed centrally by experts in pediatric epilepsy. If available, prior clinically obtained video‐EEG or video recordings may be used to confirm that the events in question are seizures if the seizure semiology has remained the same. If no clinically obtained recordings are available, then they will be obtained as study procedure before randomization. The clinically observable FOS documented by video‐EEG or video will be the events of interest for the study's primary endpoint.

In the pretreatment baseline period, titration phase, and maintenance period, the above clinically observable seizures will be recorded in seizure diaries by caregivers.

Eligible subjects who meet the study entry criteria and whose treatment regimen is at steady state at enrollment will begin a prospective baseline period during which they will continue to receive their prescribed ASMs. ASM doses must be stable for ≥4 half‐lives, and no dose change of >20% will be permitted within two weeks of enrollment. New concomitant ASMs, aside from study drug, may not be added during the trial.

During the baseline period, seizures recorded in the diary will be reviewed by the investigator to confirm that they correspond to the previously defined index seizure semiologies. Other seizure types should be recorded in the diary, but will not be used for determination of the primary study endpoint. The duration of the baseline period will be defined by the subject's seizure frequency. An example of how this may be done is presented in Table 2.

**Table 2:** Variable baseline duration is defined according to the subject's seizure burden

Baseline seizure frequency	Duration of baseline
≥1 seizure per day	7 d
Less than daily and >weekly seizures	14 d
Less than weekly but ≥3 seizure days per month	28 d

Once the baseline has ended, the total number of index seizures during baseline will be the individualized “N”. The time elapsed from end of study drug titration to the occurrence of a number of seizures equal to that subject's “N” will serve as the subject's individualized prespecified endpoint (Table 3).

**Table 3:** Sample determination of the individualized, prespecified endpoint^a^

Day 1:4 seizures	
Day 2:2 seizures	
Day 3:1 seizure	
Day 4:3 seizures	
Day 5:2 seizures	
Day 6:1 seizure	
Day 7:3 seizures	
Baseline seizure frequency: ≥1 seizure per day	Duration of baseline: 7 d
Number of seizures during baseline: 16	Individualized, prespecified endpoint: N = 16 seizures

^a^ This subject has daily seizures; therefore, the baseline period is 7 d. The total seizure count during the baseline was 16 seizures. After the subject has completed the titration phase (after randomization) and has 16 seizures (this subject's individualized prespecified endpoint), they will exit the treatment period of the study.

**Figure 1:**
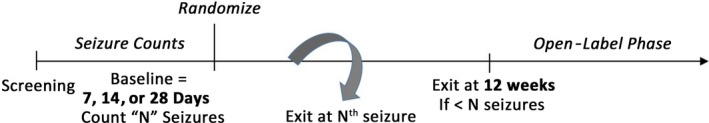
Time‐to‐event clinical trial protocol overview

Subjects who continue to meet study entry criteria at the end of the baseline period will be randomly assigned to double‐blind treatment with investigational study drug (one or more doses) or placebo. After randomization and titration (as appropriate for the specific drug to be assessed), subjects will enter the maintenance treatment phase. Subjects will remain in the maintenance treatment phase until they reach their individualized prespecified endpoint. Subjects who have not reached the individualized prespecified endpoint will continue in the double‐blind phase for up to 12 weeks (Figure 1). Efficacy will be evaluated based on time (days) elapsed from randomization to reaching the individualized prespecified endpoint of “N” seizures.

## 4 STATISTICAL DESIGN, DATA COLLECTION, AND ANALYSIS CONSIDERATIONS

The proposed trial design outlined here will encourage more providers, child neurologists, families, and patients to consider the enrollment of this young population leading most likely to a better recruitment rate. In general, the proposed design will tend to have lower power than a standard six‐ or eight‐week baseline design looking at changes in seizure frequency—because of less overall monitoring time in both the baseline and postrandomization periods. This likely will increase sample size relative to a standard design, but it should offset the difficulties in enrollment that plague current designs to more than compensate for any increase in sample size. Researchers will need to think carefully about the variable baseline lengths with respect to their understanding of the drug under study and the specifics of the planned population. For instance, the 7‐day baseline may not be appropriate if a drug has moderate efficacy, has a longer titration, or slower onset of action. To improve on power, variability can be reduced by actions such having standardized training for the caregiver(s) who document the seizures of interest, for instance, in identifying and recording seizure (eg, recording clusters).

Analysis of the time to Nth seizure can utilize most standard survival analysis methods such as Cox proportional hazards models. The typical model will need a factor for the baseline strata used in the design with the option of including baseline seizure rate for improved precision. The data from any studies in older children or even adults could be used with Bayesian methods. Such an analysis can enhance the “power” of the study, but it requires that those studies and the new study satisfy the compatibility requirement known as “exchangeability.” It would also require reanalyzing the previous studies as time to Nth seizure studies.^17^, ^18^

We developed this design to address the urgent need for infant focal epilepsy, but the design could also be considered for use in other epilepsy syndromes, including generalized epilepsies and pediatric syndromes such as Dravet and Lennox‐Gastaut syndrome.

## 5 CONCLUSIONS

Difficulty of successfully performing clinical trials in children with early‐life epilepsies creates a pressing ethical issue: The incidence of epilepsy is highest among young children, but there is a striking paucity of data upon which clinicians can rely to make informed treatment decisions in these patients. As a result, most children with early‐life epilepsies are prescribed off‐label medications without adequate evidence of efficacy and safety. Given that the safest way to initiate treatment with a new medication is through an efficient clinical trial, there is an urgent need to redesign protocols to improve efficiency in order to make them acceptable to clinicians, investigators, families, and regulatory agencies.

In the study design used in the 1990s and early 2000s, change in the number of FOS with or without clinical signs recorded on 48‐ to 72‐hour video‐EEG between baseline and posttreatment served as the primary outcome measure.^11^, ^12^, ^13^, ^14^, ^15^ Such an approach is onerous for patients and families, confounded by the inability to assess clinical signs during some EEG seizures, complicated by EEG artifacts which can mimic seizures, prone to sampling error, and not generalizable to clinical practice. Use of informed seizure diaries combines the rigor of accurate seizure identification and classification via video‐EEG with a clinically relevant seizure diary count by caregivers. Seizure diaries informed by prior EEG, video, and video‐EEG are practical, efficient, rigorous, and generalizable to clinical practice.

Our proposed design would allow the inclusion of many more infants/young children with FOS than traditionally designed trials. Prior trial designs only allowed inclusion of patients with a high seizure frequency and there were limitations in maintaining them in the trial for the baseline and the treatment period. The new design allows duration of placebo exposure to be based on the infants’ seizure burden and response (or lack of response) to treatment with the new ASM. Infants with lower seizure burden may now be included while they would not have been previously due to the requirement of video‐EEG‐recorded seizures within 2‐3 days of monitoring. The proposed design would reflect the real‐world treatment of infants with FOS. With potentially increased study‐eligible patient populations, we anticipate that the novel protocol design is likely to be completed much more efficiently than recent clinical trials of ASMs in very young populations. Ultimately, this would enhance clinical care through efficient provision of rigorous evidence to guide treatment decisions in early‐life epilepsies.

## CONFLICT OF INTEREST

S Auvin receives salary support from Université de Paris and Assistance Publique—Hôpitaux de Paris. Stéphane Auvin is Associate Editor for Epilepsia. He is an investigator on research grants awarded to Robert‐Debré University Hospital from Advicenne Pharma, EISAI, UCB Zogenix; he has received travel expenses or consulting fee from Arvelle, Advicenne Pharma, Biocodex, Biomarin, Eisai, GW Pharma, Novartis, Nutricia, Shire, UCB Pharma, Vitaflo, Zogenix. J. French receives NYU salary support from the Epilepsy Foundation and for consulting work and/or attending Scientific Advisory Boards on behalf of the Epilepsy Study Consortium for Acadia, Adamas, Addex, Aeonian, Alexza, Anavex, Arvelle Therapeutics, Inc, Axcella Health, Axovant, Biogen, Biomotiv/Koutif, Blackfynn, Bloom Science, Bridge Valley Ventures, Cavion, Cerebral Therapeutics, Cerevel, Clinilabs, Concert Pharmaceuticals, Covance, Crossject, CuroNZ, Eisai, Empatica, Encoded, Engage Therapeutics, Epitel, GW Pharma, Idorsia, Impax, Ionis, J&J Pharmaceuticals, Marinus, MonosolRx, Neurelis, Neurocrine, Novartis, Otsuka Pharmaceutical Development, Ovid Therapeutics Inc, Pfizer, Pfizer‐Neusentis, Praxis, Redpin, Sage, Sancillio, Shire, SK Life Sciences, Springworks, Stoke, Sunovion, Supernus, Takeda, UCB Inc, Ultragenyx, Upsher Smith, Vyera, West Therapeutic Development, Xenon, Xeris, Zogenix, Zynerba.J. French has also received research grants from Biogen, Cavion, Eisai, Engage, GW Pharma, Lundbeck, Neurelis, Ovid, SK Life Sciences, Sunovion, UCB, and Zogenix as well as grants from the Epilepsy Research Foundation, Epilepsy Study Consortium, and NINDS. She is on the editorial board of Lancet Neurology and Neurology Today. She is scientific officer for the Epilepsy Foundation for which NYU receives salary support. She has received travel reimbursement related to research, advisory meetings, or presentation of results at scientific meetings from the Epilepsy Study Consortium, the Epilepsy Foundation, Adamas, Arvelle Therapeutics, Inc, Axovant, Biogen, Blackfynn, Crossject, CuroNz, Eisai, Engage, Idorsia, Neurelis, Novartis, Otsuka, Ovid, Pfizer, Redpin, Sage, SK Life Science, Sunovion, Takeda, UCB, Ultragenyx, Zynerba. Dr Dlugos receives salary support from NIH, Commonwealth of Pennsylvania Department of Health, Pediatric Epilepsy Research Foundation, and The Epilepsy Study Consortium; he is an investigator on research grants awarded to CHOP from Zogenix, Greenwich Biosciences, UCB, Brain Sentinel, Neurelis, Pfizer, Q‐State, USL, Aquestive, Sage, Bio‐Pharm, Insys, SK Life Sciences, Biogen, and Encoded Therapeutics; he has received travel expenses for protocol development or investigator meetings from Marinus, Ovid/Takeda, Ultragenyx, USL, Pfizer, and Zogenix. Dr Knupp receives research support from West pharmaceuticals, Zogenix, and the Pediatric Epilepsy Research Foundation. She has received consulting funds from Zogenix and Greenwich Biosciences. Dr Perucca received speakers/consultancy fees from Amicus Pharma, Arvelle, Biogen, Eisai, GW Pharma, Sanofi, Sun Pharma, Takeda, UCB Pharma, and Xenon Pharma. His contribution to this work was partly supported by a European Union grant to the EpiCARE European Reference Network. Dr Arzimanoglou receives salary support from the University Hospitals of Lyon (HCL). His work is also partly supported by the European Union grant to the EpiCARE European Reference Network. He has a mission of Editor‐in‐Chief for the ILAE educational journal Epileptic Disorders and of Associate Editor for the European Journal of Paediatric Neurology. He is an investigator on research grants awarded to HCL, France and Sant Joan de Déu Hospital Barcelona from the Caixa Foundation and UCB; he has received travel expenses or consulting fees from Advicenne Pharma, Biomarin, Eisai, GW Pharma, Shire, UCB Pharma, Zogenix. Ed Whalen, PhD, is a full‐time employee of Pfizer Inc He holds Pfizer stock and receives stock as part of his compensation. Dr Shellhaas receives research support from PCORI, NIH, and the Pediatric Epilepsy Research Foundation. She serves as a consultant to the Epilepsy Study Consortium and receives royalties from UpToDate for authorship of topics related to neonatal seizures. We confirm that we have read the Journal's position on issues involved in ethical publication and affirm that this report is consistent with those guidelines.
